# A study protocol for a randomized controlled trial to assess the effects of Baduanjin exercise on balance impairment in patients with cervical dystonia

**DOI:** 10.3389/fneur.2025.1633287

**Published:** 2025-11-11

**Authors:** Shun Fan, Jixuan Wang, Shiying Zhang, Chuanfeng Tao, Qiao Huang, Yinghui Jin, Huanan Li, Jingui Wang

**Affiliations:** 1 First Teaching Hospital of Tianjin University of Traditional Chinese Medicine, Tianjin, China; 2 National Clinical Research Center for Chinese Medicine Acupuncture and Moxibustion, Tianjin, China; 3 Center for Evidence-Based and Translational Medicine, Zhongnan Hospital of Wuhan University, Wuhan, China; 4 Haihe Laboratory of Modern Chinese Medicine, Tianjin, China

**Keywords:** Baduanjin, cervical dystonia, balance, gait, protocol, randomized controlled trial

## Abstract

**Background:**

Patients with cervical dystonia (CD) commonly exhibit varying degrees of impaired balance, abnormal gait, and increased fear of falling. However, Botulinum toxin—the standard of care—does not substantially improve balance or gait. Baduanjin, a traditional Chinese exercise, improves balance in several neurological disorders, yet its effects in CD remain unknown. This protocol describes a randomized controlled trial evaluating the effects of Baduanjin exercise on balance impairment in patients with CD.

**Methods:**

This prospective randomized controlled trial enrolls CD patients with impaired balance. Eligible participants are randomly allocated (1:1) to either a Baduanjin intervention group or a control group without Baduanjin. The Baduanjin group undergoes a 20-week training program. The primary outcome is balance function, evaluated through the Berg Balance Scale. Secondary outcomes include standing static balance ability assessed by the Zebris Stance Analysis FDM System, fall risk conducted using the Timed Up & Go test, gait-related data assessed by the Zebris Gait Analysis FDM System, the severity of CD assessed by the Toronto Western Spasm Rating Scale, and the anxiety status of patients assessed by the Hamilton Anxiety Rating Scale. The entirety of the data are collected at the baseline, 2, 6, 12, and 20 weeks. A two-way mixed analysis of variance (ANOVA) or generalized estimating equations are used to assess the effectiveness of Baduanjin.

**Discussion:**

This protocol is the first randomized controlled design to evaluate Baduanjin for balance and gait in cervical dystonia, combining objective instrumented outcomes with validated clinical scales and a pragmatic supervised-to-home regimen, thereby providing decision-relevant evidence.

**Clinical trial registration:**

http://itmctr.ccebtcm.org.cn/zh-CN/Home/ProjectView?pid=fce76993-0978-484e-ba50-c097b35805c7, ITMCTR2024000240.

## 1 Introduction

Cervical dystonia (CD) is among the most common focal dystonias in clinical practice. It is characterized by involuntary contractions of neck muscles (e.g., sternocleidomastoid, trapezius, splenius), leading to abnormal postures or movements and often accompanied by pain (1). This condition significantly impairs patients’ normal work and quality of life, exacerbating social burdens (2, 3). The prevalence of CD is approximately 8.9 per 100,000 individuals, with 70 to 90% of patients exhibiting symptoms between the ages of 40 and 60 (4, 5).

The etiology and pathogenesis of CD remain incompletely understood. The academic community generally agrees that CD involves lesions in multiple brain structures, including cortex, cerebellum, thalamus, and brainstem, are implicated (6). Animal models have demonstrated that abnormal cerebellar activity is a contributing factor to dystonia. Such aberrant cerebellar activity often leads to impaired balance in patients. Balance issues are common in neurological disorders, and a systematic review (7) has shown that abnormal cerebellar activity frequently causes muscle spasms, balance deficits, and gait abnormalities in patients with conditions such as CD and Parkinson’s disease. The effect of abnormal head posture on physical function in CD remains unclear (8). A study revealed that, compared to healthy adults, CD patients often exhibit deficits in balance, gait, and stepping reactions, along with a heightened fear of falling (9). A subset of CD patients also experience tremors, which exacerbate their balance and gait impairments (10, 11). Additionally, individuals with CD have a substantially higher lifetime risk of psychiatric disorders, affecting over 90% of patients (12, 13), and anxiety is among the most common comorbidities (14).

Although botulinum toxin remains the primary treatment for CD (15, 16), it has not been proven to relieve motor manifestations or significantly change balance control or walking ability (17). Consequently, there is an urgent imperative to explore intervention strategies aimed at enhancing the balance capabilities of CD patients.

Comparatively, Baduanjin is an effective method for balance training and is even superior to simple walking (18). Systematic reviews have shown that Baduanjin improves motor performance, equilibrium, and gait in individuals with Parkinson’s disease (19), promotes lower-limb motor recovery and daily activity in stroke patients (20), and reduces anxiety symptoms in individuals with mental disorders (21). The exercise is easy to learn and enjoys broad popularity nationwide in China (22).

Although Baduanjin has demonstrated benefits for balance dysfunction in other neurological conditions, there is a dearth of randomized controlled trial (RCT) data evaluating its effects in CD. Therefore, this protocol aims to investigate the potential of Baduanjin to enhance balance function and its impact on gait in patients with CD.

## 2 Methods and analysis

### 2.1 Objectives

1. The main purpose of this study is to evaluate the effects of a 20-week Baduanjin program versus no Baduanjin on balance in patients with CD.
2. The secondary purpose is to evaluate the effects of Baduanjin on gait, disease severity, and anxiety.
3. This study also evaluates the safety of Baduanjin in patients with CD.

### 2.2 Hypothesis

1. Based on usual care, the addition of Baduanjin exercises results in better balance outcomes in the Baduanjin group.
2. The Baduanjin group experiences improvements in gait, disease severity, and anxiety.
3. Baduanjin is expected to be a safe therapeutic approach for patients with CD.

### 2.3 Study design

**Figure 1:**
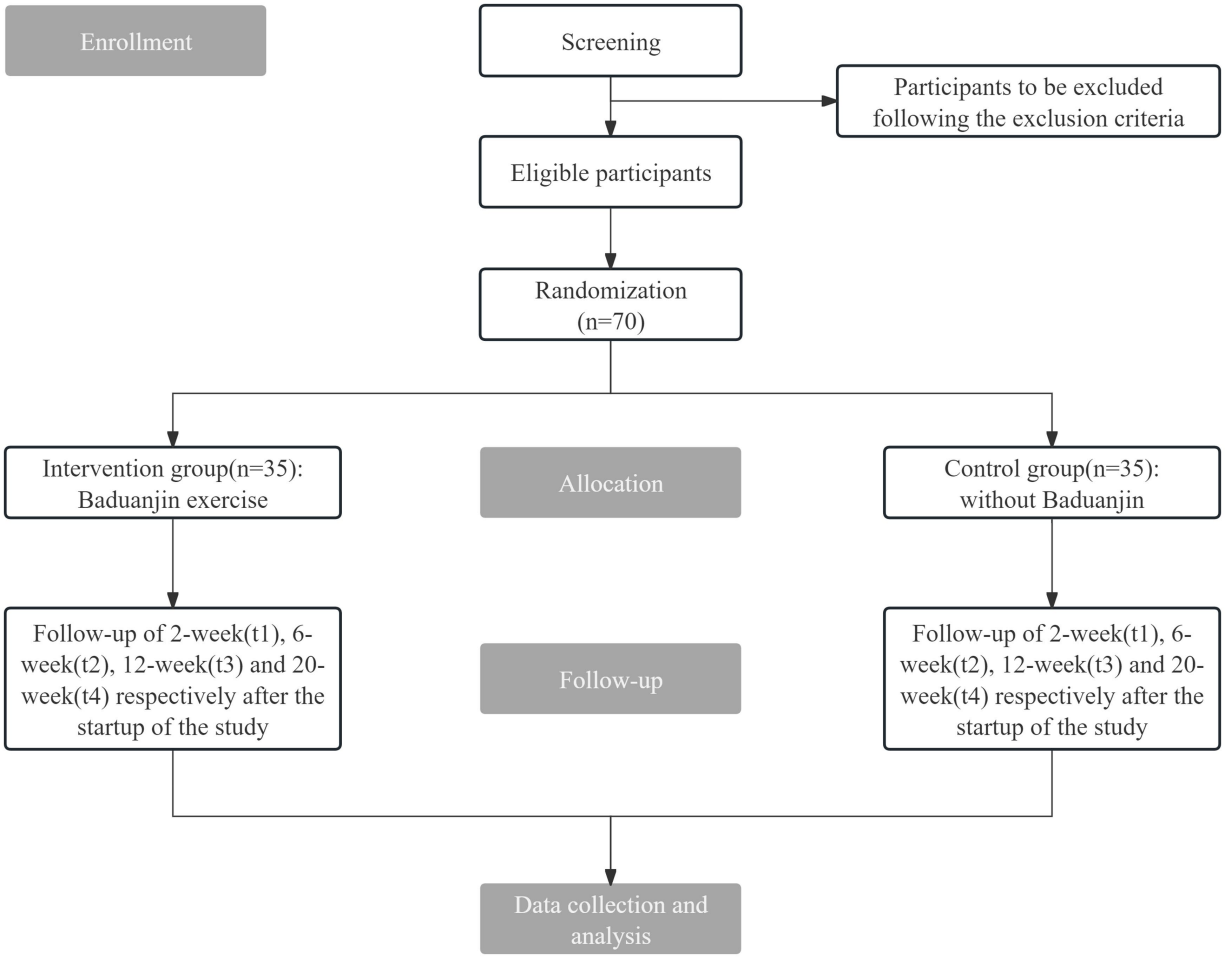
The flow chart of this trial.

This study is designed as a prospective, non-blinded, parallel-group randomized controlled trial. Seventy eligible patients are randomized (1:1) to the Baduanjin intervention or the control without Baduanjin. The hypothesis is tested using significance testing, and the study design follows a parallel RCT framework. Outcomes are assessed at baseline and at 2, 6, 12, and 20 weeks. The schedule of enrolment, interventions, and assessments is outlined in Table 1. The study flow is illustrated in Figure 1. The study protocol adheres to the Standardized Protocol for Information Retrieval for Interventional Trials 2025 Statement (Supplementary material 1) (23).

**Table 1:** The schedule of enrolment, interventions, and assessments.

Time point	Enrolment		Post-randomisation (20 weeks)				Close-out
	–	0	t_1_	t_2_	t_3_	t_4_	t_x_
	3 days to 0		2 weeks ± 3 days	6 weeks ± 3 days	12 weeks ± 3 days	20 weeks ± 3 days	
Enrolment							
Eligibility screen	X						
Informed consent	X						
Randomization		X					
Interventions							
Baduanjin		X	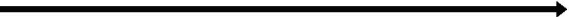				X
Control group		X	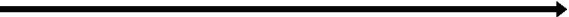				X
Assessments							
BBS	X		X	X	X	X	X
SSBA		X	X	X	X	X	X
TUG test		X	X	X	X	X	X
Gait analysis		X	X	X	X	X	X
TWSTRS		X	X	X	X	X	X
HAMA		X	X	X	X	X	X
Safety assessment	Keep track						

BBS, Berg Balance Scale; SSBA, standing static balance ability; TUG, The Timed Up & Go; TWSTRS, The Toronto Western Spasmodic Torticollis Rating Scale; HAMA, Hamilton Anxiety Rating Scale.

### 2.4 Participants

Participants have been recruited since April 30, 2025, from the CD specialty ward of the First Teaching Hospital of Tianjin University of Traditional Chinese Medicine, and recruitment is currently ongoing. Recruitment information has been disseminated through social media (WeChat official account) and hospital posters. The diagnostic, inclusion, exclusion, and removal criteria are defined as follows.

#### 2.4.1 Diagnostic criteria

We base the diagnosis on the core clinical features of CD as proposed in the 2013 revised classification of dystonia (24): involuntary, sustained contractions of neck muscles causing twisting/repetitive movements or abnormal postures. In addition, clinical symptoms that resemble dystonia but are not characteristic of dystonic disorders are differentiated according to the same guideline (25).

Given the lack of specific diagnostic tests, the diagnosis of CD relies primarily on the identification of patient symptoms by experienced clinicians. For patients with CD who are difficult to diagnose, ancillary examinations are performed, such as electromyography to assist in clinical judgment and brain Magnetic Resonance Imaging (MRI) to exclude other potential conditions.

#### 2.4.2 Inclusion criteria

1. Fulfill the diagnostic criteria for primary CD.
2. Score less than 54 on the Berg Balance Scale (BBS) (26).
3. Age between 18 and 65 years, gender not specified.
4. No structural damage or abnormalities are detected in brain MRI scans.
5. Have not received botulinum toxin injections or undergone motor training within the 3 months before enrollment.
6. Provide informed consent by signing the relevant document.

#### 2.4.3 Exclusion criteria

1. Have undergone treatment with deep brain stimulation, the modified Foerster-Dandy procedure, and selective muscle removal for spasticity.
2. Pregnant or lactating women.
3. Patients with serious diseases of the heart, brain, lungs, and other critical organs might make such individuals unsuitable for Baduanjin.
4. Patients with cognitive dysfunction caused by conditions such as Parkinson’s disease or others.
5. Patients with diseases affecting balance, such as cerebral infarction or chorea.
6. Patients with conditions affecting gait, such as lumbar disc herniation or knee osteoarthritis.

#### 2.4.4 Removal criteria

1. Occurrence of any serious adverse event.
2. Withdrawal of informed consent.
3. Determination by the principal investigator (PI) that exclusion from the study is necessary. Participants exhibiting the following conditions are mandatorily excluded from the per-protocol set (PPS): Obvious or serious deviation from the protocol during implementation, or poor compliance, defined as completing less than 60% of the prescribed exercise duration.

### 2.5 Sample size

No prior study has evaluated Baduanjin on balance in CD. Therefore, we estimated the sample size using data from previously designed similar studies. It was found that Baduanjin combined with usual care increased the BBS score by 3.7 points compared with usual care alone (27). Standard deviations were *σ*_1_ = 5.37 (Baduanjin) and *σ*_2_ = 4.32 (control). According to the formula for two independent samples with unequal variances:


$$n_{1}=n_{2}=\frac{{\left(z_{1-\frac{\alpha }{2}}+z_{1-\beta }\right)}^{2}(\sigma_{1}^{2}+\sigma_{2}^{2})}{\delta^{2}}$$

With 80% power and a two-sided *α* = 0.05, and allowing for 20% missing data, the required sample size is 35 participants per group.

### 2.6 Informed consent

The study invites all CD patients who comply with the inclusion and exclusion criteria. The written informed consent form (ICF) describes the study purpose, procedures, potential benefits/risks, compensation, data protection, and withdrawal rights. The ICF must be thoroughly reviewed by each patient. The patients’ inquiries are carefully addressed by the research team. The ICF is signed by both parties to verify that the patient has fully understood the study protocol. Prior to conducting any interventions, it is essential to acquire a written ICF from every participant. Randomization occurs only after signed ICFs are obtained.

### 2.7 Randomization and allocation

Randomization and allocation are independently conducted by a researcher who does not participate in data capture or analysis. This process uses the R package “randomizr” within R Project V.4.4.2, with a seed set to 123, and a 1:1 allocation ratio (35 per group), ensuring reproducibility of the randomization results. The outcomes of the block randomization are sealed in opaque envelopes. Each time a new patient is enrolled in the study, an envelope is opened to determine the participant’s group assignment based on the enclosed allocation plan.

### 2.8 Blinding

In view of the particularity of the intervention, blinding is not applied to doctors or patients. Outcome assessors and statisticians are blinded to allocation to minimize bias and ensure role separation among investigators, assessors, and analysts.

### 2.9 Interventions

During the initial two-week hospitalization, patients in both groups receive usual care provided by the specialized CD ward. This care includes five times a week of neck muscle massage and traditional Chinese medicine fuming for the neck. And at the same time, health education on balance function is provided to the patients. After discharge, we continue to provide regular health education to both groups of patients in order to prevent overestimation of the intergroup effect caused by the Hawthorne effect and to ensure that both groups feel equally attended to.

#### 2.9.1 Control group

The control group will not undertake any exercise training and will be asked to maintain routine daily activity. At 20 weeks post-enrollment, patients are advised to perform Baduanjin training, and corresponding guidance is provided.

#### 2.9.2 Baduanjin exercise group

Patients undergo supervised training in the ward for 2 weeks (5 days per week) during their 14-day stay from admission to discharge. Researchers offer instructions on performing Baduanjin exercises to the participants and distribute corresponding manuals and demonstration videos. We engage Baduanjin instructors with over 5 years of teaching experience to conduct in-ward training sessions, correct patients’ movements, and fine-tune the exercise intensity based on individual physical conditions. The intervention utilizes the Baduanjin regimen promulgated by the General Administration of Sport of China in 2003:

1. Both Hands Lift the Sky to Regulate the Triple Burner.
2. Drawing the Bow Left and Right to Shoot the Eagle.
3. Separating Heaven and Earth to Harmonize the Spleen and Stomach.
4. Looking Backward to Eliminate Five Stresses and Seven Injuries.
5. Swaying the Head and Shaking the Tail to Dissipate Heart Fire.
6. Hands Grasp the Feet to Strengthen the Kidneys and Waist.
7. Clenching Fists with Fierce Eyes to Boost Strength.
8. Seven Bounces on the Heels to Cure All Ailments.

The practice of Baduanjin requires professional instruction, with coaches first breaking down and demonstrating each movement to help patients gradually master the techniques before proceeding with systematic training. All Baduanjin sessions are conducted by the same instructor. Sessions is about 50 min in duration, including 10 min of warm-up, 30 min of Baduanjin training, and a 10-min stretching phase. Following the two-week in-ward program, patients continue the same routine at home for 18 weeks, exercising 3 days per week. Consequently, the total exercise duration per week amounts to 150 min. This regimen is selected because previous Baduanjin trials commonly reported durations ranging from 6 to 24 weeks, with total weekly exercise times between 120 and 300 min (28).

Baduanjin is a traditional Chinese qigong regimen composed of eight standardized movements, characterized by moderate intensity. Each movement is easily learned and memorized, offering benefits in regulating various organ functions. The principles of treatment in traditional Chinese medicine, such as “movement generates yang energy,” emphasize enhancing yang energy through exercise to adjust bodily functions and improve conditions, aligning with the concepts of “exercise as medicine” proposed by the American College of Sports Medicine and the American Medical Association.

The first section stretches the limbs and torso, strengthening the neck and upper arm muscles. The second section, involving isometric upper limb movements and squatting, enhances lower limb strength, increases venous return, and improves cardiorespiratory function. The third section adjusts the core muscles of the waist through upward and downward stretching of the upper limbs. The fourth section, by rotating the neck, stretches the muscles on either side and stimulates the carotid arteries to improve cerebral blood flow. The fifth section shifts body weight from side to side, improving local circulation and lower limb strength. The sixth section, involving bending forward and pressing down with both hands, strengthens muscles related to the spine and enhances spinal stability and flexibility. The seventh section, focusing on clenching fists and forcefully striking, improves concentration and reaction speed, relieving stress and regulating mood through powerful movements. The eighth section, balancing on tiptoes, strengthens calf muscles and enhances balance, with the shock from heel contact traveling up the spine to the brain, particularly stimulating intervertebral spaces and nerve branches from the spinal cord to regulate internal organ function.

### 2.10 Quality control

Effective quality control is capable of improving patient compliance. To encourage active participation and enhance adherence to the intervention plan, researchers employ several strategies:

1. Detailed Explanation of Benefits: Patients are thoroughly informed of the advantages associated with being randomized to the Baduanjin group.
2. Coach-Driven Motivation: Instructors foster enthusiasm for the Baduanjin training sessions by maintaining an engaging and motivating atmosphere during the physical exercises.
3. Daily Reminders via WeChat: Research assistants use a WeChat group to remind participants to follow their respective exercise regimens. Participants are prompted to upload daily exercise video data, allowing researchers to monitor the correctness of their exercise postures.
4. Consistent Engagement: Regular face-to-face meetings and phone calls are maintained to sustain participants’ interest. Additionally, educational materials and lectures are provided to facilitate ongoing communication, expressing continual support and gratitude to the participants.
5. Flexible Scheduling: To accommodate potential conflicts between exercise routines and participants’ daily lives, researchers adjust the study schedule as needed to ensure feasibility and convenience for the participants.

Participants who complete the program, regardless of their group assignment, receive a cash reward. Adherence is calculated weekly based on the uploaded video data, considering the total exercise duration.

### 2.11 Outcomes

#### 2.11.1 Primary outcome

The primary outcome is balance, assessed with the Berg Balance Scale (BBS). This comprehensive assessment instrument was developed by Katherine Berg and her team at New York University (29). The BBS comprises 14 distinct tasks, with each task scored based on the participant’s performance, utilizing a scoring range of 0–4 points per task, culminating in a maximum total score of 56 points. Previous research has demonstrated a strong correlation between impaired trunk control ability and balance dysfunction in patients with CD, with an average BBS score of 53 (26). Lower scores indicate progressively poorer balance function.

#### 2.11.2 Secondary outcomes

1. The Zebris Stance Analysis FDM system (30) is used to assess standing static balance ability. Patients undergo two tests in standing posture with eyes open and closed, respectively, each lasting 30 s with a sampling rate of 100 Hz. The system records the trajectory of the center of plantar pressure for each condition and calculates the center of pressure path length and the area of the 95% confidence ellipse during quiet stance.
2. The Timed Up and Go (TUG) is used to assess mobility and fall risk in cervical dystonia (CD). It records the time to rise from a chair, walk 3 m, turn, return, and sit. A completion time of less than 10 s is generally considered normal, indicating that the subject has good balance and gait.
3. The Zebris Gait Analysis FDM system (31) is used for the collection and analysis of gait parameters, including walking speed, cadence, step length, step width, gait cycle, double support ratio, and gait symmetry index. The gait test lasts for 1 min with a sampling rate of 100 Hz. The gait symmetry index is defined as the difference between the right and left side gait line length. A value closer to 0 indicates higher symmetry and better balance (32).
4. The Toronto Western Spasm Rating Scale (33) is used to assess the severity of CD patients. The scale comprises three main components: the severity of torticollis, disability level, and pain score, with a total possible score of 85. A higher total score indicates greater severity of torticollis.
5. The Hamilton Anxiety Rating Scale (34) is used to evaluate anxiety symptom severity. The instrument contains 14 items, each scored 0–4, for a cumulative range of 0–56; a total score of 7 or above suggests anxiety, and increasing scores reflect increasing severity.

#### 2.11.3 Safety assessment

During the implementation of the study, any adverse events experienced by participants are documented. Research assistants complete adverse event forms and report them to the research team. We compute the adverse event rate for both groups using the formula: the number of CD patients who experienced adverse events/total number of enrolled CD patients × 100%. Supervisors and the research team evaluate the adverse events, assessing the time of occurrence, symptoms, severity (mild, moderate, severe, potentially life-threatening, or fatal), duration, management measures, and outcomes. They determine the correlation with the trial treatment (not related, possibly related, or definitely related), sign the forms, and date them. The adverse event rates are recorded. Severe adverse events should be immediately reported to the ethics committee. If a participant suffers harm as a direct result of trial participation, they are provided with compensation and necessary medical treatment in accordance with national regulations and institutional policies.


$$\text{Adverse event rate}(\%)=\frac{\begin{array}{c} \text{number of}\;\mathrm{CD}\;\text{patients}\;\mathrm{who}\;\\ \text{experienced adverse events} \end{array}}{\begin{array}{c} \text{total number of}\;\\ \text{enrolled}\;\mathrm{CD}\;\text{patients} \end{array}}\times 100\%$$

### 2.12 Data analysis

All statistical tests are two-sided, and a *p*-value ≤0.05 is considered statistically significant. Missing data are imputed using the multiple imputation method, and modified intention-to-treat analysis is employed. The analysis follows the intention-to-treat (ITT) principle, and the results from the PPS are used for sensitivity analysis. Participants whose cumulative exercise time reaches at least 60% but does not exceed 120% of the prescribed duration are classified into the PPS. The primary efficacy endpoint of this study is assessed at Week 20.

Means ± standard deviations are used to describe normally distributed continuous variables, while medians and interquartile ranges, along with minimum and maximum values, are used for non-normally distributed data. Frequency rates (%) are used to describe categorical data. For continuous variables, between-group comparisons at the same time point are conducted using two-independent samples *t*-test or Mann Whitney U test, as appropriate. Categorical data are analyzed using the chi-square test. If there are significant differences in sociological or clinical characteristics between groups, covariance analysis is performed for adjusted testing.

For repeated measures data, within-group differences across time points are analyzed using one-way repeated measures ANOVA. If the data do not meet the assumptions of normality and sphericity, the Friedman test is used, and Bonferroni correction is used for multiple comparisons. For the interaction between group and time, two-way mixed ANOVA is used. If the data do not meet the normality assumption, generalized estimating equations are employed to evaluate the effectiveness of the intervention. The incidence of adverse events is analyzed using chi-square test. If formal statistical comparisons between groups are not possible due to a lack of effectiveness, descriptive statistics are used to tabulate and summarize adverse events.

Statistical analyses are conducted using R Project V.4.4.2. The stats package is used for the normality test, homogeneity of variance test, independent sample t-test, Mann Whitney U test, chi-square test, Friedman test, and Bonferroni correction. The car package is used for the sphericity test and covariance analysis. The Afex package is used for one-way ANOVA and two-way mixed ANOVA. The Lme4 package is used for generalized estimating equations, and the multiple imputation method is performed using the mice package.

### 2.13 Data collection and management

Sociological information on patients is collected through the hospital information system. Clinical outcome measures are obtained from paper research medical records and then entered into the specialized electronic database developed by the Supercomputing Center of Tianjin University.^1^ Two data managers independently perform double data entry and verification. This database complies with current security standards and is protected by a dedicated password. Data entry personnel enter the data, but do not have access to view it. Only statistical personnel are allowed to access and view the data. Upon completion of this study, the de-identified individual participant-level data are made available through the Individual Participant Data Sharing Platform^2^ to ensure confidentiality and support further research.

A Data Monitoring Committee (DMC) is not to be established for this study because the intervention poses minimal risk to participants and the study duration is relatively short. The PI is responsible for weekly data monitoring and safety oversight.

## 3 Discussion

Although there is now a considerable amount of evidence indicating that Baduanjin can improve the balance disorders caused by neurological diseases (28, 35, 36), However, no study has directly proven that Baduanjin can improve the balance ability of patients with CD. CD is significantly associated with balance dysfunction and abnormal gait (8, 37). Most intervention methods can only relieve the spasm symptoms of the patients’ neck muscles and rarely pay attention to the patients’ motor symptoms. Baduanjin is a Chinese qigong form with a history of more than 800 years. It has attracted more and more people around the world and also aroused extensive attention in the medical field. Compared with balance exercise movements, Baduanjin is easier to learn and does not require much physical strength. It consists of eight movements, each of which combines hardness and softness. During the practice, patients with CD need to effectively combine breathing adjustments with movements. For a long time, Baduanjin has not only been widely practiced among the public as a way to strengthen the body but has also gradually attracted attention in the medical field.

Previous studies have confirmed that Baduanjin can improve balance ability through multiple pathways (36, 38, 39). An RCT showed that Baduanjin can improve gait reaction ability by enhancing lower-limb muscle strength (36). A systematic review shows that Baduanjin can activate the precuneus and right median cingulate in the brain to influence the regulation of limb movement (38). Baduanjin training can improve the knee joint proprioception of individuals with knee osteoarthritis, thereby enhancing the patients’ postural stability and reducing the risk of falls (39).

Accordingly, we designed a rigorous, parallel-group RCT that focuses on the effects of Baduanjin on balance dysfunction in patients with CD. The trial is intended to fill the therapeutic gap whereby standard botulinum toxin care fails to meaningfully improve balance in CD. The intervention is pragmatically structured to transition from supervised in-hospital training to standardized home-based practice, thereby enhancing the real-world feasibility and scalability of this low-cost, easily implemented exercise modality. To mitigate assessment bias for the primary endpoint, we prespecified objective, instrumented outcome measures. Collectively, these design features strengthen internal validity and implementation relevance. This trial will provide direct evidence for patients with balance impairment regarding the improvement of balance ability in patients with CD by Baduanjin.

There are some limitations to this protocol. First, as this is the first RCT protocol focusing on the impact of Baduanjin exercise on the balance and gait of patients with CD, we rely on data from similar studies to calculate the sample size. Considering that CD is a relatively rare disease, the sample size is small, but it has sufficient statistical power. Secondly, it is difficult to quantify the exercise intensity. Baduanjin is a traditional health-preserving exercise. There are large individual differences in exercise intensity, which are affected by various factors such as physical condition, movement proficiency, and the degree of effort of the practitioners. In this study, although a unified practice plan was formulated, it is difficult to accurately measure the actual exercise intensity of each patient. Finally, considering that some patients in the experimental group may continue to practice Baduanjin after the end of the trial, and the control group needs compensatory practice guidance, this protocol did not set up a follow-up to observe the long-term efficacy of Baduanjin. Despite the above challenges, we still strive to conduct the trial in accordance with the protocol.

## Footnotes

^1^ https://172.16.0.64:1555/#/DASHBOARD

^2^ http://www.medresman.org.cn/login.aspx
